# The Role of Serum Metabolomics in Distinguishing Chronic Rhinosinusitis With Nasal Polyp Phenotypes

**DOI:** 10.3389/fmolb.2020.593976

**Published:** 2021-01-12

**Authors:** Shaobing Xie, Hua Zhang, Yongzhen Liu, Kelei Gao, Junyi Zhang, Ruohao Fan, Shumin Xie, Zhihai Xie, Fengjun Wang, Weihong Jiang

**Affiliations:** ^1^Department of Otolaryngology Head and Neck Surgery, Xiangya Hospital of Central South University, Changsha, China; ^2^Hunan Province Key Laboratory of Otolaryngology Critical Diseases, Changsha, China

**Keywords:** chronic rhinosinusitis with nasal polyps, eosinophil, metabolomics, metabolites, biomarker

## Abstract

**Background:** Chronic rhinosinusitis with nasal polyps (CRSwNP) is a heterogeneous disease characterized by different clinical features and treatment responsiveness. This study aimed to compare the serum metabolomics profiles between eosinophilic CRSwNP (eCRSwNP) and non-eosinophilic CRSwNP (neCRSwNP) and healthy controls (HC) and explore objective biomarkers for distinguishing eCRSwNP before surgery.

**Methods:** Serum samples were collected from 33 neCRSwNP patients, 37 eCRSwNP patients, and 29 HC. Serum metabolomics profiles were investigated by ultra-high-performance liquid chromatography–mass spectrometry.

**Results:** The analysis results revealed that neCRSwNP, eCRSwNP, and HC exhibited distinctive metabolite signatures. In addition, eCRSwNP could be distinguished from neCRSwNP referring to their serum metabolic profiles, and the top ten different metabolites were citrulline, choline, linoleic acid, adenosine, glycocholic acid, L-serine, triethanolamine, 4-guanidinobutyric acid, methylmalonic acid, and L-methionine, which were related to several most important pathways including arginine and proline metabolism; glycine, serine, and threonine metabolism; linoleic acid metabolism; and purine metabolism. Among these distinctive metabolites, citrulline, linoleic acid, adenosine, and 4-guanidinobutyric acid showed good predictabilities, and the serum levels of citrulline, linoleic acid, and adenosine were significantly correlated with tissue eosinophil (T-EOS) percentage and T-EOS count.

**Conclusion:** eCRSwNP patients exhibited discriminative serum metabolic signatures in comparison with neCRSwNP patients and HC. These results suggested that metabolomics profiles contributed to understanding the pathophysiological mechanisms of CRSwNP and distinguishing its phenotypes

## Introduction

Chronic rhinosinusitis (CRS) is a common inflammatory disease characterized by inonasal mucosa paranasal sinuses with nasal blockage, rhinorrhea, post-nasal discharge, and olfactory dysfunction (Yao et al., 2017). Previous studies reported that CRS affected ~5.5–28% of the general population worldwide, and the prevalence still continues to increase (Chitsuthipakorn et al., 2018; Grayson et al., 2019; Li et al., 2019; Yao et al., 2019). Based on the presence or absence of nasal polyp, CRS is grouped into chronic rhinosinusitis with nasal polyps (CRSwNP) and chronic rhinosinusitis without nasal polyps (CRSsNP) (Bayar Muluk et al., 2019; Qing et al., 2019; Hoy, 2020). Due to disease heterogeneity, CRSwNP is further classified into eosinophilic CRSwNP (eCRSwNP) and non-eosinophilic CRSwNP (neCRSwNP), and these two phenotypes have obviously different disease characteristics, treatments, and prognosis (Ho et al., 2018; Fujieda et al., 2019; Yao et al., 2020). In comparison with neCRSwNP, eCRSwNP exhibits more serious disease symptoms, a higher rate of comorbid asthma, poorer treatment response, and a higher risk of recidivism (Ho et al., 2018; Hoy, 2020). Thus, a pre-operative examination that could discriminate eCRSwNP from neCRSwNP was pivotal to develop personalized treatments and follow-up. However, endotyping of CRSwNP is extremely challenging for rhinologists because of a lack of objective approaches. Therefore, it is urgently needed to develop an objective indicator or biomarker to distinguish CRSwNP phenotypes before surgery that can improve the prognosis and long-term management strategies.

Metabolomics is a burgeoning omics technology which provides opportunities to establish a powerful exploratory tool for monitoring disease status and help to expound the pathogenesis of diseases (Kelly et al., 2017; Turi et al., 2018; Spertini, 2020). Recent studies utilized metabolomics analysis to evaluate the metabolic signature in airway inflammatory diseases, such as asthma (Reisdorph and Wechsler, 2013), allergic rhinitis (Ma et al., 2020), pneumonia (Ning et al., 2018), and chronic obstructive pulmonary disease (Adamko et al., 2015) and identified several biomarkers and major metabolic pathways which might improve the understanding of these disorders and develop novel therapy target. However, no previous study has employed metabolomics to analyze metabolites and metabolic pathway changes in the serum of CRSwNP patients and explore objective biomarkers to distinguish eCRSwNP before surgery.

Therefore, we aimed to evaluate the serum metabolic signatures of CRSwNP and explore the association between the metabolite differences and CRSwNP phenotypes. In the present study, ultra-high-performance liquid chromatography–mass spectrometry (UHPLC-MS) was utilized to investigate serum metabolic profiles in eCRSwNP, comparing with neCRSwNP and healthy control (HC). Linear regression analysis was conducted to assess the correlation between different metabolites and tissue eosinophil (T-EOS) percentage and T-EOS count in CRSwNP patients.

## Materials and Methods

### Participants and Settings

We recruited 70 consecutive patients with CRSwNP from June 2018 to October 2018 in our tertiary clinic. CRSwNP was diagnosed referring to the guidelines of the European Position Paper on Rhinosinusitis and Nasal Polyps 2012 (Fokkens et al., 2012). Exclusion criteria are as follows: (1) other nasal or sinus diseases, such as fungal sinusitis, allergic rhinitis, cystic fibrosis, aspirin-exacerbated respiratory disease, and tumor; (2) treatment including antibiotics, oral or systemic corticosteroids, immunotherapy, or anti-allergic drugs 4 weeks before the surgery; (3) inflammatory, septic diseases or autoimmune diseases; (4) age <18 years or >75 years old; (5) severe heart, kidney, or other organ dysfunction; and (6) pregnant condition. All CRSwNP patients received routine preoperative examination, including blood tests, nasal endoscopy, computed tomography (CT) or magnetic resonance imaging (MRI), chest X-rays, and electrocardiography. All participants scored their nasal symptoms by utilizing the widely accepted visual analog scale (VAS) as previously described (Zhu et al., 2020). Preoperative CT score was recorded using the Lund–Mackay staging system (Lund and Mackay, 1993). A total of 29 age- and gender-matched healthy volunteers with no evidence of rhinitis or rhinosinusitis, diabetes mellitus, or inflammatory or autoimmune conditions were enrolled as healthy controls (HC).

### Diagnosis of eCRSwNP and neCRSwNP

During the surgery, nasal polys were obtained from all patients with CRSwNP, then immersed in 10% formalin and embedded with paraffin wax. The embedded tissues were sectioned at 5-μm thickness and were stained with hematoxylin and eosin (H&E) for the visualization of eosinophils. The numbers of eosinophils, lymphocytes, neutrophils, and plasma cells were counted in 10 randomly selected high-power fields by two observers who were blinded to the clinical data. eCRSwNP was diagnosed when the tissue eosinophils (T-EOS) percentage was higher than 10% of total inflammatory cells, otherwise defined as neCRSwNP (Hu et al., 2012; Zhong et al., 2020).

### Serum Sample Collection and Preparation

Fasting peripheral whole blood from CRSwNP patients and HC were collected with vacuum blood collection tubes in the morning. The blood samples were centrifuged at 1,200 g for 10 min at 4°C within 1 h of venipuncture. The serum samples were collected and stored at −80°C. Serum samples were thawed on ice and vortexed thoroughly. The serum samples were mixed with 300 μL methanol and vortexed for 30 s and incubated at −40°C for 1 h and centrifuged at 12,000 g for 10 min at 4°C. 100 μL of supernatant was transferred to a fresh tube vial for UHPLC-MS analysis (Dunn et al., 2011; Naz et al., 2014). The quality control (QC) sample was utilized as previous described to assess the stability and reliability of the analytical system (Liu et al., 2016).

### UHPLC-MS Analysis

The untargeted metabolomic analysis was performed by utilizing a 1,290 Infinity series UHPLC System (Waters Corporation, Milford, MA, USA). The mobile phase was composed of 25 mmol/L ammonium acetate in water was applied as phase A, and 25 mmol/L ammonia in acetonitrile was used as phase B. The analysis procedure was processed as previously described (Zhao et al., 2019). The Triple TOF 6600 mass spectrometry (AB Sciex, Boston, MA, USA) was used to obtain spectra data, and the acquisition software (Analyst TF 1.7, AB Sciex, Framingham, MA, USA) continuously evaluated the full-scan survey MS data. In each cycle, the most intensive 12 precursor ions (intensity > 100) were chosen for MS/MS at collision energy (CE) of 30 eV. The cycle time was 0.56 s. Electrospray ionization (ESI) source conditions were set as previous study described (Liu et al., 2016; Zhao et al., 2019).

### Data Processing and Analysis

MS raw data (.wiff) files were converted to the mzXML format by Proteo Wizard and processed by R package XCMS V3.2. The process includes peak deconvolution, alignment, and integration. Peak extraction and alignment were performed by Proteo Wizard and analyzed by R package as previous study described (Kuhl et al., 2012; Zhao et al., 2019). Metabolites identification refers to the In-house MS2 database. The processed data was exported to SIMCA (Version 14.1, Umetrics, Umea, Sweden) for multivariate analysis. Orthogonal partial least squares-discriminant analysis (OPLS-DA) was conducted to identify the major latent metabolites in the data matrix (Yang et al., 2020). The quality of the models was validated by R2Ycum (goodness of fit) and Q2cum (goodness of consistency). Meanwhile, the 200 permutations of cross-test were conducted to reduce the risk of overfitting and possibilities of false-positive findings. Metabolites contributing were calculated based on the variable importance for project (VIP) values (VIP > 1.0) and *P*-values (*P* < 0.05) (Wang et al., 2018). A volcano plot was presented to project the metabolic regulations of the remarkable shifts in metabolites. The receiver operating characteristic (ROC) analysis was applied to the serum data to assess the performance of potential biomarker, and the area under the curve (AUC) was calculated to evaluate the sensitivity and specificity. To identify associated metabolic pathways, the pathway analysis was conducted using MetaboAnalyst 3.0.

### Statistical Analysis

Continuous variables are described as mean ± standard deviation (SD). When the variables distributed normally, one-way analysis of variance (ANOVA) or Student's *t*-test was used, otherwise Kruskal–Wallis H test or Mann–Whitney *U*-test was performed. Discontinuous variables were described as number (percentage) and compared using Chi-square test. To evaluate the correlation between different metabolites and T-EOS percentage and T-EOS count in CRSwNP patients, Spearman's correlation analyses were performed. Significant difference was accepted when *P*-value < 0.05. All statistical analyses were conducted on SPSS statistics software version 23.0 (IBM, Chicago, IL, USA).

## Results

### Baseline Characteristics of All Subjects

**Figure 1:**
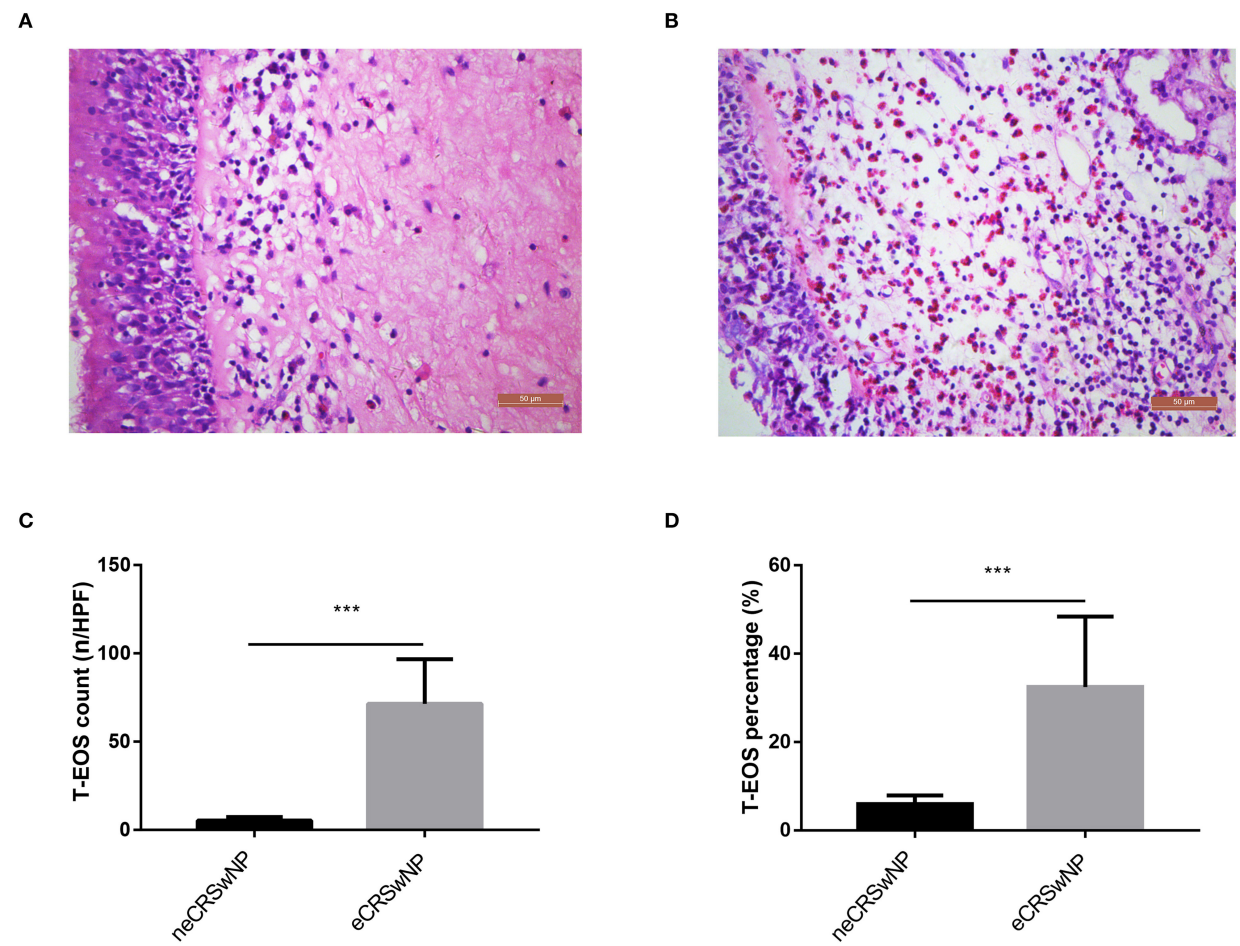
Representative H&E staining of neCRSwNP and eCRSwNP. **(A)** neCRSwNP. **(B)** neCRSwNP. **(C,D)** Comparison of T-EOS percentage and T-EOS count per HPF between neCRSwNP and eCRSwNP. Mann–Whitney U test was utilized. eCRSwNP, eosinophilic chronic rhinosinusitis with nasal polyps; neCRSwNP, non-eosinophilic chronic rhinosinusitis with nasal polyps; T-EOS, tissue eosinophil; H&E, hematoxylin and eosin; HPF, high-power field. ***P < 0.001.

Demographic and clinical characteristics of all subjects are listed in the Table 1. Among 70 CRSwNP patients, 33 (47.14%) patients were identified as eCRSwNP, and the other 37 (52.86%) patients were defined as neCRSwNP. Compared to the HC and neCRSwNP groups, the eCRSwNP group showed higher levels of blood eosinophil (B-EOS) count and B-EOS percentage (all *P* < 0.001). However, no statistical difference was observed in age, gender, rate of smoking, drinking, and BMI among three groups, and VAS score and Lund–Mackay score between the neCRSwNP and eCRSwNP groups. Typical histological findings of neCRSwNP and eCRSwNP are exhibited in Figures 1A,B. The T-EOS count and percentage in the eCRSwNP patients were significantly higher than those in the neCRSwNP (all *P* < 0.001, Figures 1C,D).

**Table 1:** Clinical characteristics of subjects.

**Variables**	**HC (*n* = 29)**	**neCRSwNP (*n* = 33)**	**eCRSwNP (*n* = 37)**	***P*-value**
Age (years), mean ± SD	28.5 ± 8.5	32.8 ± 12.2	35.1 ± 14.8	0.103
Gender (male/female), *n*	14/15	18/15	19/18	0.885
Smoking (yes/no), *n*	10/19	17/16	19/18	0.306
Drinking (yes/no), *n*	7/22	8/25	12/25	0.673
BMI (kg/m^2^), mean ± SD	22.2 ± 1.8	22.7 ± 1.8	22.3 ± 1.5	0.570
B-EOS counts (10^6^/L), mean ± SD	81.2 ± 24.0	174.7 ± 84.4	405.8 ± 159.3	<0.001
B-EOS percentage, %	1.2 ± 0.8	2.2 ± 1.0	4.5 ± 1.2	<0.001
VAS score, mean ± SD	–	5.5 ± 1.5	5.9 ± 1.7	0.300
Lund-Mackay score, mean ± SD	–	18.8 ± 3.9	18.2 ± 3.8	0.499

*eCRSwNP, eosinophilic chronic rhinosinusitis with nasal polyps; neCRSwNP, non-eosinophilic chronic rhinosinusitis with nasal polyps; HC, healthy control; SD, standard deviation; BMI, body mass index; B-EOS, blood eosinophil; VAS, visual analog scale*.

### Metabolomic Signatures of neCRSwNP vs. HC

**Figure 2:**
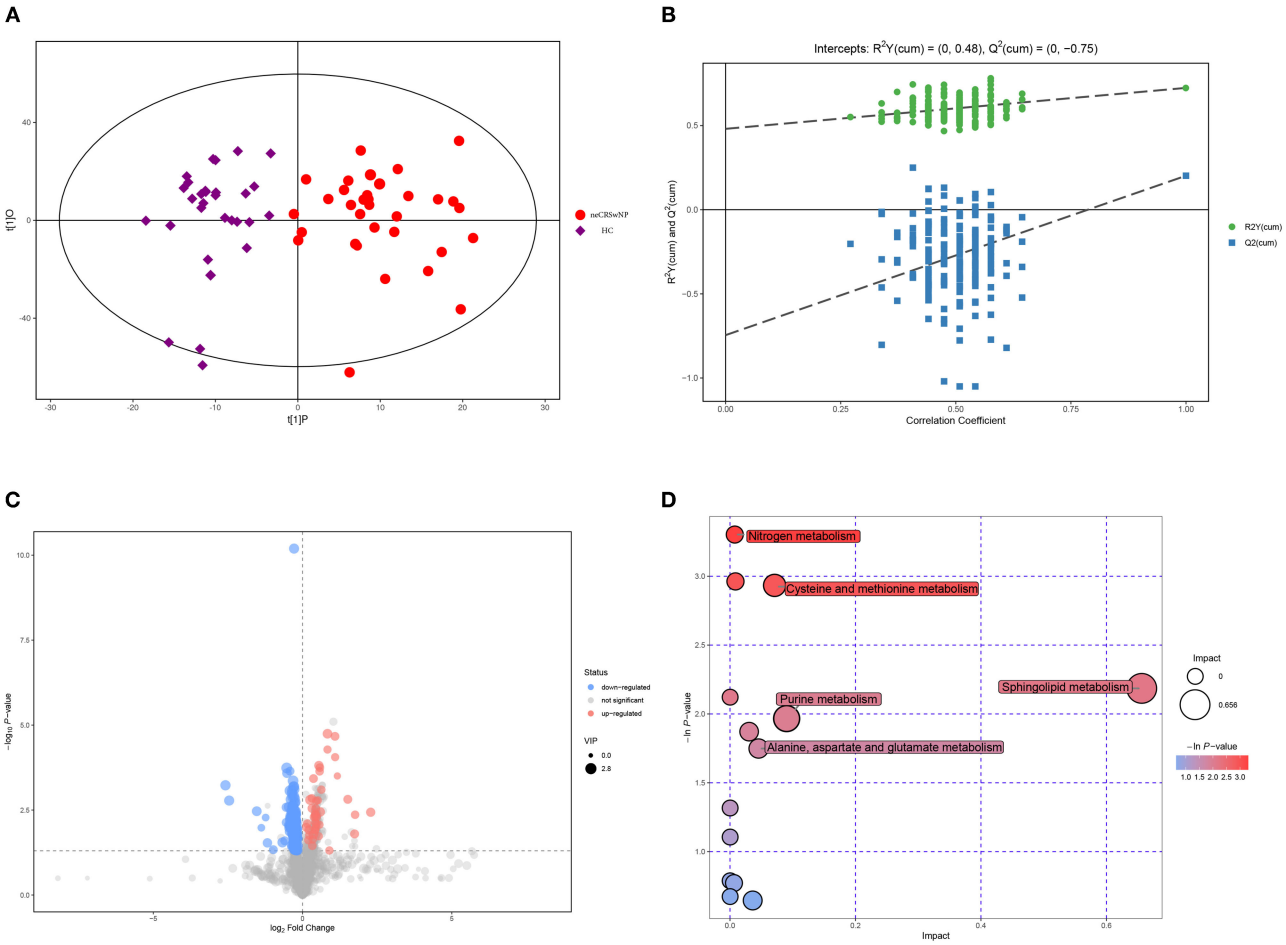
Metabolomic analysis of serum samples of neCRSwNP and HC. **(A,B)** OPLS-DA model and permutation test of the OPLS-DA model. **(C)** Volcano plot. **(D)** Metabolic pathway bubble chart. neCRSwNP, non-eosinophilic chronic rhinosinusitis with nasal polyps; HC, healthy control; OPLS-DA, orthogonal partial least square-discriminate analysis.

The OPLS-DA model exhibited a clear and distinctive clustering between neCRSwNP and HC (Figure 2A), R2X (cum), R2Y (cum), and Q2 were 0.236, 0.724, and 0.202, respectively. The OPLS-DA model was then assessed by permutation analysis, and all permuted R2s were below or around 0.6 and all permuted Q2s were below 0, which means that all R2s and Q2s are lower than the original on the right (Figure 2B). Thus, this suggests that the model fittings were valid and predictive. The potential differential metabolites were selected referring to the contribution of VIP (VIP > 1 and *P* < 0.05). Finally, a total of 20 metabolites including 11 upregulated and nine downregulated for distinguishing neCRSwNP from HC were detected by UHPLC-MS analysis and they are shown in Figure 2C. In addition, metabolic pathway analysis results showed that cysteine and methionine metabolism and purine metabolism were the major involved metabolic pathways (Figure 2D).

### Metabolomic Signatures of eCRSwNP vs. HC

**Figure 3:**
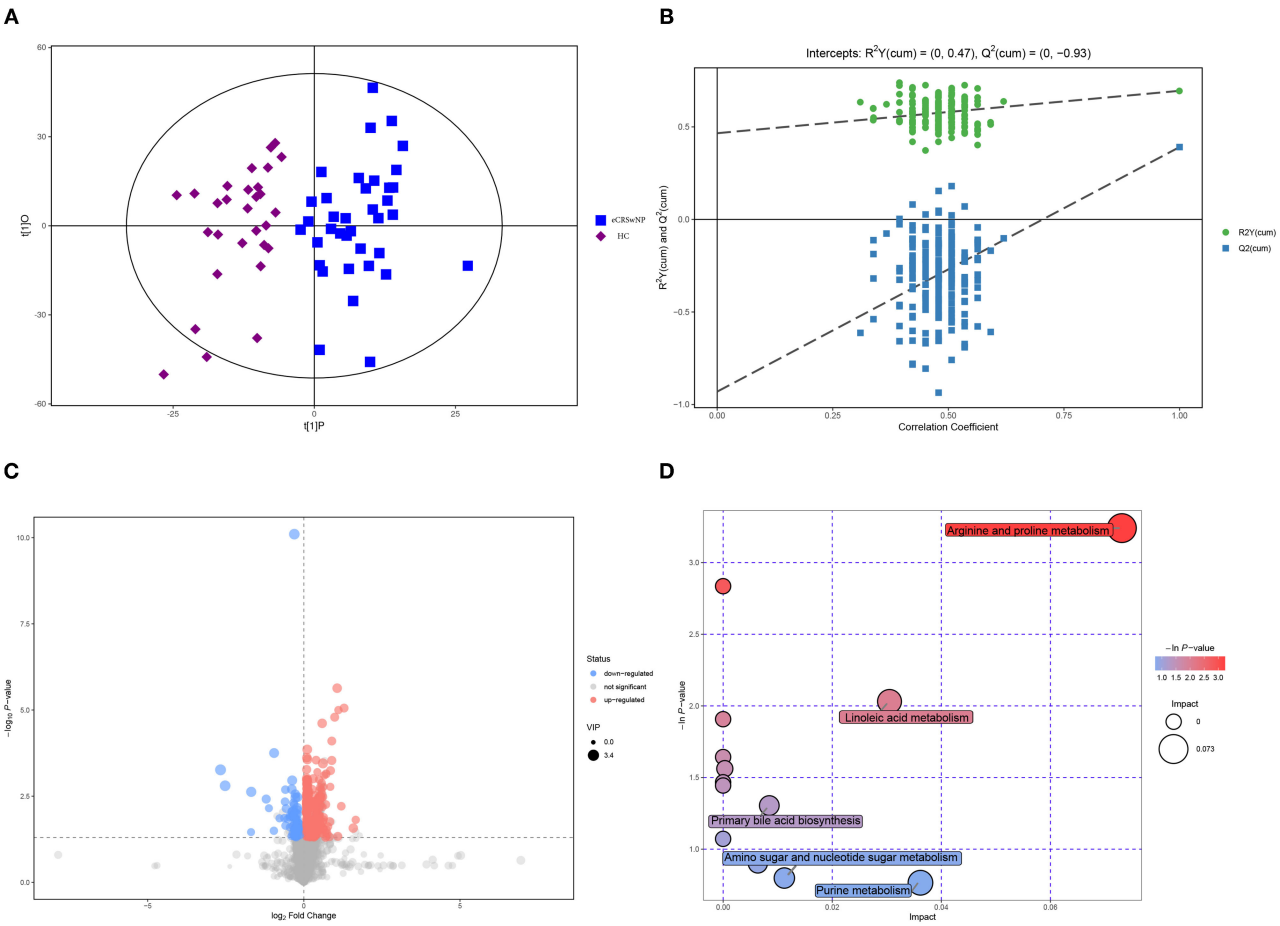
Metabolomic analysis of serum samples of eCRSwNP and HC. **(A,B)** OPLS-DA model and permutation test of the OPLS-DA model. **(C)** Volcano plot. **(D)** Metabolic pathway bubble chart. eCRSwNP, eosinophilic chronic rhinosinusitis with nasal polyps; HC, healthy control; OPLS-DA, orthogonal partial least square-discriminate analysis.

Figure 3A shows that eCRSwNP patients were distinguished from HC based on serum metabolic profiles. In the OPLS-DA model, R2X (cum), R2Y (cum), and Q2 were 0.254, 0.695, and 0.391, respectively, and the model was assessed by permutation analysis, and analysis results suggested that the model fittings were valid and predictive (Figure 3B). Compared to HC, 49 metabolites were expressed at significantly different concentrations in the eCRSwNP group including 39 upregulated and 10 downregulated (Figure 3C). The most affected pathways including arginine and proline metabolism and linoleic acid metabolism are displayed in the Figure 3D.

### Metabolomic Signatures of eCRSwNP vs. neCRSwNP

**Figure 4:**
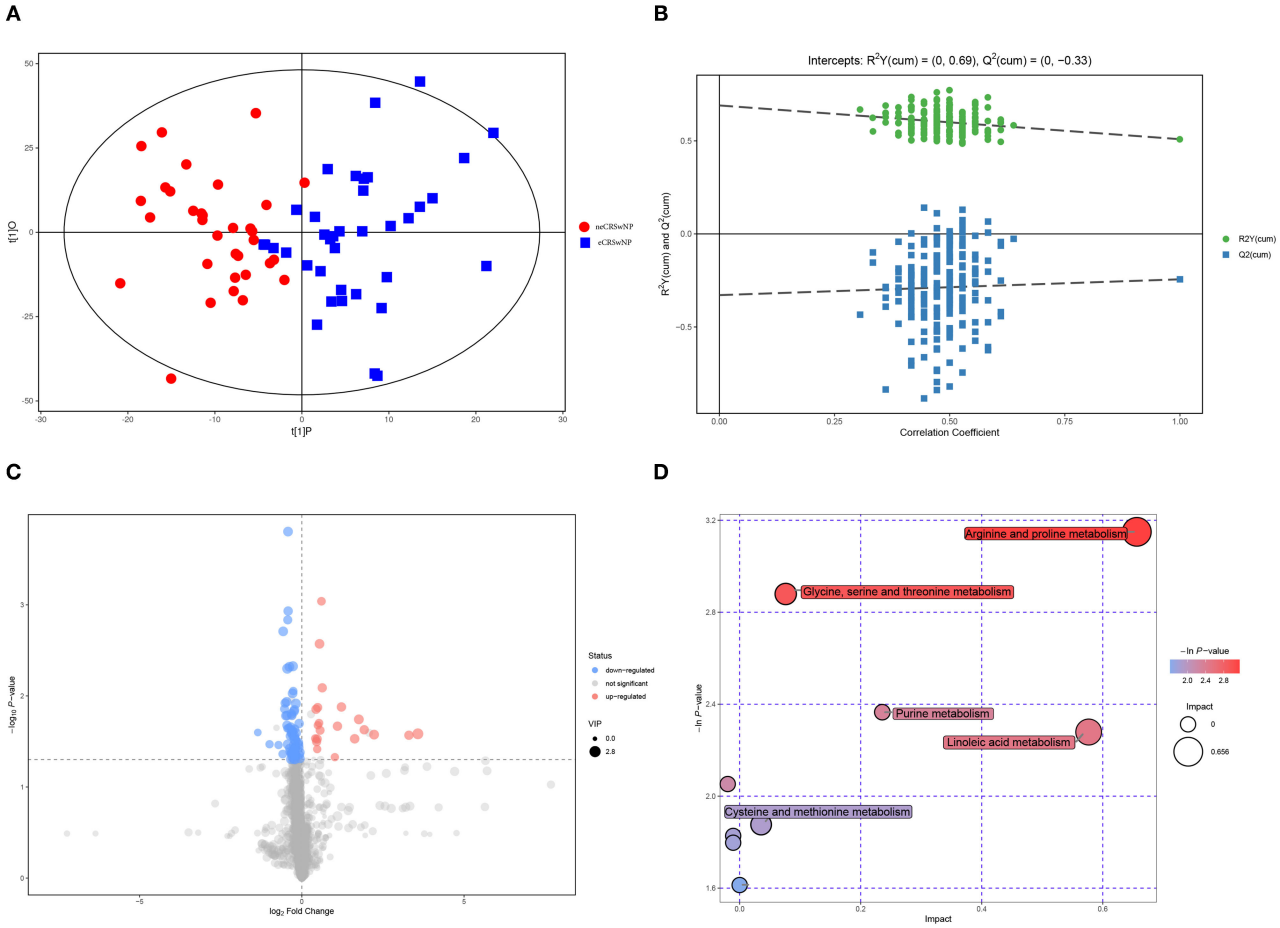
Metabolomic analysis of serum samples of neCRSwNP and neCRSwNP. **(A,B)** OPLS-DA model and permutation test of the OPLS-DA model. **(C)** Volcano plot. **(D)** Metabolic pathway bubble chart. neCRSwNP, non-eosinophilic chronic rhinosinusitis with nasal polyps; eCRSwNP, eosinophilic chronic rhinosinusitis with nasal polyps; OPLS-DA, orthogonal partial least square-discriminate analysis.

**Figure 5:**
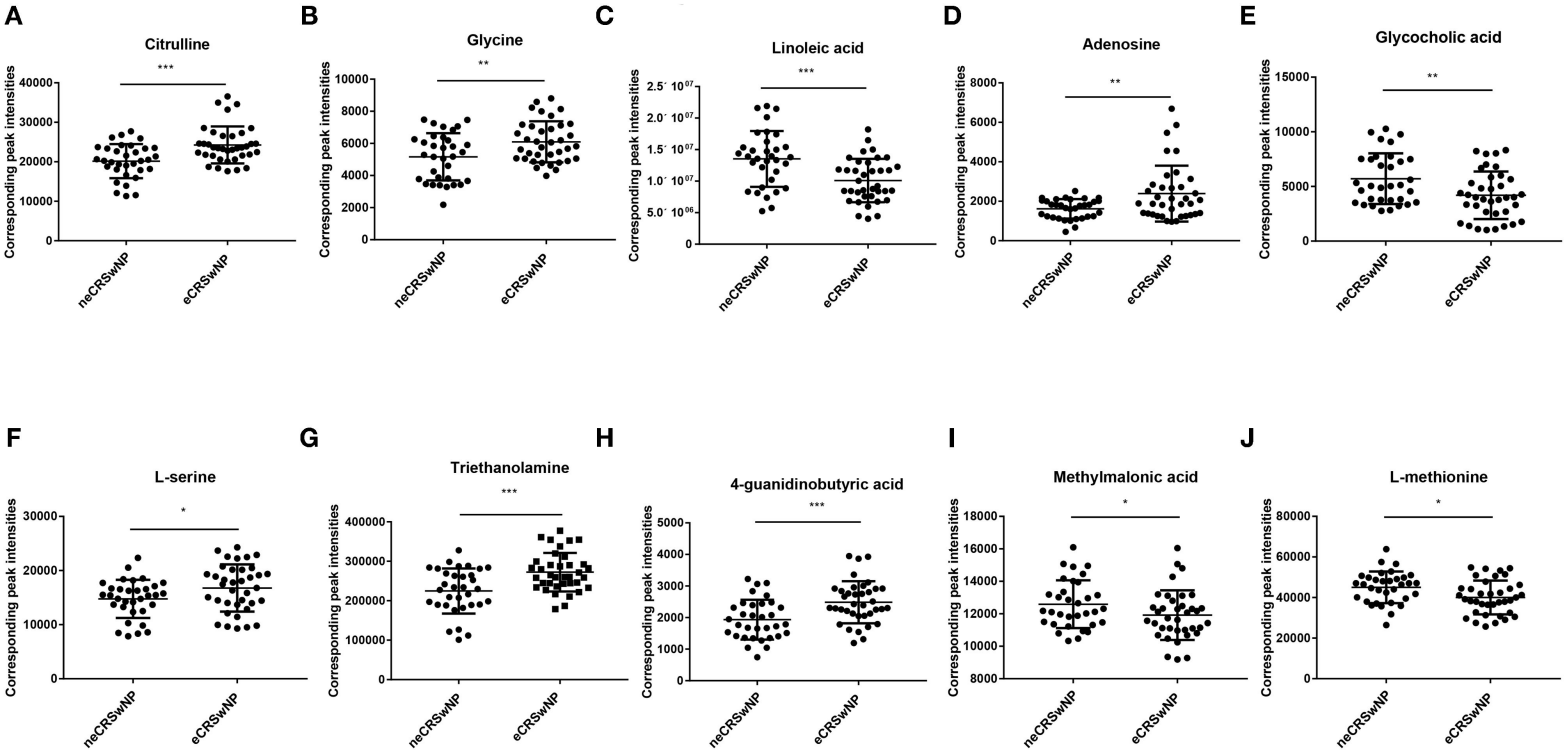
Top 10 most discriminant metabolites in their relative levels in neCRSwNP group and neCRSwNP group. neCRSwNP, non-eosinophilic chronic rhinosinusitis with nasal polyps; eCRSwNP, eosinophilic chronic rhinosinusitis with nasal polyps. Mann–Whitney *U*-test was used for the statistical analysis. *P < 0.05, **P < 0.001, ***P < 0.001.

Figure 4A exhibits that serum metabolomic profiles of eCRSwNP patients had significantly different serum metabolomics profiles in comparison with neCRSwNP patients. In the OPLS-DA model, R2X (cum), R2Y (cum), and Q2 were 0.215, 0.509, and 0.244, respectively. The permutation analysis results demonstrated that the discriminating models were reliable (Figure 4B). In the eCRSwNP group, 24 metabolites were observed at different levels including 11 upregulated and 13 downregulated in comparison with the neCRSwNP group (Figure 4C). The most important pathways including arginine and proline metabolism; glycine, serine, and threonine metabolism; purine metabolism; and linoleic acid metabolism are displayed in the Figure 4D. Results of top 10 potential discriminant metabolites are displayed in the Table 2, and their relative serum concentrations between two groups are comparatively shown in Figure 5. The ROC curves of these distinctive metabolites are depicted in Supplementary Figure 1, and analysis results are shown in Supplementary Table 1. Citrulline, linoleic acid, adenosine, and 4-guanidinobutyric acid exhibited good accuracy for distinguishing eCRSwNP (AUC > 0.7), and they were included in Spearman's correlation analysis to explore their association with the severity of eosinophils infiltration in the nasal polys. The serum levels of citrulline and adenosine were positively correlated with T-EOS percentage and T-EOS count, while linoleic acid levels were negatively correlated with T-EOS percentage and T-EOS count (Supplementary Figure 2).

**Table 2:** Top 10 metabolites discriminating eCRSwNP from neCRSwNP.

**Metabolites**	**VIP**	***P***	**FC**	**AUC**	**Pathways**
Citrulline	2.73	<0.001	4.11	0.791	Arginine and proline metabolism
Glycine	2.47	0.008	0.36	0.544	Glycine, serine and threonine metabolism
Linoleic acid	2.13	<0.001	0.41	0.823	Linoleic acid metabolism
Adenosine	2.08	<0.001	2.48	0.902	Purine metabolism
Glycocholic acid	1.94	0.005	2.06	0.627	Primary bile acid biosynthesis
L-Serine	1.90	0.024	0.52	0.615	Glycine, serine and threonine metabolism
Triethanolamine	1.84	<0.001	1.93	0.524	Glycerophospholipid metabolism
4-Guanidinobutyric acid	1.80	0.002	2.37	0.809	Arginine and proline metabolism
Methylmalonic acid	1.79	0.036	1.77	0.672	Pyrimidine metabolism
L-methionine	1.72	0.010	0.62	0.690	Cysteine and methionine metabolism

*eCRSwNP, eosinophilic chronic rhinosinusitis with nasal polyps; neCRSwNP, non-eosinophilic chronic rhinosinusitis with nasal polyps; VIP, variable importance for project; FC, fold change; AUC, area under the curve*.

## Discussion

CRSwNP is a complex disease with persistent inflammation in the nasal and sinonasal mucosa, and its physiopathologic mechanisms are poorly clarified (Yamada et al., 2019). Considering the heterogeneity, CRSwNP is divided into eCRSwNP and neCRSwNP, and these two phenotypes have distinctive clinical and pathologic features, drug sensitivity, prognosis, and recurrence rate (Sivrice et al., 2020). Thus, discriminating eCRSwNP from neCRSwNP through a simple and reliable method before surgery is important to promote the precision medicine and improve the management strategies. Up to now, tissue pathological evaluation with H&E staining is the golden standard to diagnose eCRSwNP, which is invasive and relatively subjective and inapplicable to patients who prefer non-surgical treatment (Brescia et al., 2020). Therefore, it is urgently needed to develop an easy, minimally invasive, objective, and feasible method or biomarker to identify subtypes of CRSwNP before treatment. Our study is the first one to describe an innovative application of metabolomics analysis in exploring metabolic signatures to distinguish CRSwNP phenotypes. Our analysis results showed that eCRSwNP exhibited discriminative serum metabolites and metabolic pathway in comparison with neCRSwNP and HC. These results suggested that serum metabolomics was useful for developing objective biomarkers for distinguishing eCRSwNP, and the metabolites and metabolic pathway highlighted in the present study will help us to improve the understanding of underlying pathogenesis of eCRSwNP and explore new therapeutic targets.

We firstly reported that the arginine and proline metabolism pathway was disturbed in eCRSwNP patients. Previous studies demonstrated that arginine metabolism was pivotal in the nitric oxide (NO) synthesis and associated with cellular metabolism, inflammation, and immune response (Xu et al., 2016). In a recent study, Xu et al. (2017) found that arginine metabolism was regulated in the asthma patients, and the elevated level of arginine promoted the production of fraction of exhaled nitric oxide and then aggravated asthma symptoms. In another study, the researchers reported that the concentrations of ornithine, citrulline, creatine, creatinine, and sarcosine were increased in the serum of asthma patients, and they suggested that arginine metabolism was the most crucial in the development of asthma (Quan-Jun et al., 2017). Liang et al. (2019) observed that arginine metabolism was significantly changed in the serum of commuters who exposure to automobile exhaust, and the arginine metabolism dysfunction increased oxidative stress and inflammation response and aggravated air pollution toxicity. In the present study, we also found that the serum concentrations of citrulline and 4-guanidinobutyric acid were significantly elevated in the eCRSwNP group, and the AUCs for discriminating eCRSwNP were 0.791 and 0.809, respectively, and the serum levels of citrulline were positively correlated with T-EOS percentage and T-EOS count. Citrulline and 4-guanidinobutyric acid were the downstream products of arginine metabolism, and they were proved to participate in regulating T cell proliferation and differentiation and promote inflammatory response in several diseases including asthma and allergic rhinitis (Xu et al., 2017). In addition, citrulline is a key molecule in the citrulline–arginine–NO cycle, and it has been demonstrated to maintain the high NO production and promote the cellular metabolism and the inflammation response (King et al., 2004; Xu et al., 2016). Thus, we suggested that arginine metabolism associated with the development of eCRSwNP, and citrulline could distinguish eCRSwNP and associate with the severity of eosinophils infiltration. Further studies are needed to discover the underlying mechanism.

We firstly found that linoleic acid metabolism was disturbed strongly in the serum of eCRSwNP, and the serum levels of linoleic acid were decreased in eCRSwNP patients compared to neCRSwNP patients and HC and were negatively correlated with T-EOS percentage and T-EOS count, which meant linoleic acid might be a promising biomarker for discriminating eCRSwNP and a novel therapeutic target. Recently, increasing evidence demonstrated that fatty acid metabolism played emerging roles in regulating immune responses in allergic and inflammatory diseases (Arita, 2016; Ishihara et al., 2019). A previous study reported that oleic acid could reduce the production of inflammatory cells and eosinophils in bronchial alveolar lavage fluid, and IgE in serum of mouse models, then suppressed the occurrence and development of asthma (Lee et al., 2019). Previous publications showed that polyunsaturated fatty acids could affect the functions of T cells via inhibiting its proliferation and activation, and also could suppress the activation and secretion of mast cells (Yu and Björkstén, 1998; Wang and Kulka, 2015; Arita, 2016; Radzikowska et al., 2019). Linoleic acid, a common polyunsaturated fatty acid, has been proved to be crucial in activating both autophagy and antioxidation in a synergistic feedback loop and greatly aids in the prevention and treatment of multiple inflammatory disease (Wang and Kulka, 2015; Lee et al., 2019). Therefore, we have reasons to believe that linoleic acid may play a pivotal role in the eCRSwNP and can serve as an objective indication for distinguishing CRSwNP phenotypes.

Another interesting finding was that the serum concentrations of adenosine were most indicative of distinguishing eCRSwNP and reflecting the severity of eosinophil infiltration in the nasal polys tissue. Adenosine, an endogenous purine nucleoside, can be accumulated during different physiologic and pharmacologic processes, such as hypoxia, trauma, and inflammation, and several studies suggested that it played an important role in modulating mast cell, monocytes, and T cell functions (Gomez et al., 2013; Yuryeva et al., 2015). A recent study reported that adenosine was produced in high concentrations in the serum of chronic obstructive pulmonary diseases, and the serum levels of adenosine significantly correlated with disease severity (Singh Patidar et al., 2018). Mao et al. (2020) found that the levels of adenosine were significantly increased in the plasma of chronic spontaneous urticaria and associated with disease activity, and they also observed that plasma adenosine was a promising biomarker for predicting treatment outcomes. Vass et al. (2006) demonstrated that the adenosine concentrations were elevated in the exhaled breath of allergic rhinitis and positively correlated with NO concentrations. Collectively, these studies provide a reasonable explanation for our observation of elevated adenosine in eCRSwNP and its value in distinguishing CRSwNP phenotypes.

Of note, abnormal glycine, serine, and threonine metabolism was also found in the present study, and the serum levels of glycine and L-serine were decreased in the eCRSwNP patients. Accordingly, serine is one of the crucial amino acids in the synthesis of human proteins, and L-serine, another isoform of serine, was proven to be pivotal in suppressing the production of reactive oxygen species and reducing oxidative stress in several inflammatory diseases (Rodriguez et al., 2019). A previous study reported that L-serine provided components for nerve function and exerted anti-inflammatory properties, it could relieve chronic pain in low-back and knee pain patients (Sasahara et al., 2020). Glycine has been previously demonstrated to be critical in controlling the levels of oxygen species, and it exhibits anti-inflammatory and immunomodulatory effects in several disorders (Yang et al., 2020). Alonso et al. (2016) utilized nuclear magnetic resonance to analyze the urine metabolites of several inflammatory diseases and found that the serum levels of glycine were significantly decreased. Therefore, we ultimately believed that glycine and L-serine were associated with eCRSwNP, and they might serve as novel metabolic biomarkers for discriminating CRSwNP phenotypes.

Our study has several limitations. First, the sample size is relatively small, and a validation cohort study is needed to confirm the conclusions. Second, all included patients are from single centers with the same ethnicity and region, which might limit their generalization. Third, there is a wide variation in diagnostic criteria of eCRSwNP among previous reports, and no clear criteria currently exists regarding the cutoff value, this may limit the applicability of our findings. Lastly, because CRSwNP is a nasal and sinus disease mainly characterized by local inflammation and metabolic changes, the degrees of systemic metabolic disturbance and metabolic pathway dysfunction are relatively low, and these may partly influence the accuracy and predictability of OPLS-DA and permutation models. Future studies with larger sample sizes and unified diagnostic criteria are needed to validate and strengthen our present conclusion.

In conclusion, we have demonstrated that serum metabolomics could be utilized to distinguish CRSwNP phenotypes and establish metabolic signatures which might reflect the severity of eosinophil infiltration. These results suggested that metabolomic profiles contributed to understanding the pathophysiological mechanisms of eCRSwNP.

## Data Availability Statement

The raw data supporting the conclusions of this article will be made available by the authors, without undue reservation.

## Ethics Statement

The studies involving human participants were reviewed and approved by the ethical committee of Xiangya Hospital of Central South University. The patients/participants provided their written informed consent to participate in this study.

## Author Contributions

ShaoX and HZ wrote the manuscript. HZ and YL collected the sample. KG and JZ performed the data analysis. RF and ShuX provided statistical support. ZX, FW, and WJ designed the research study. All authors reviewed the manuscript and approved the final version.

## Conflict of Interest

The authors declare that the research was conducted in the absence of any commercial or financial relationships that could be construed as a potential conflict of interest.

## Supplementary Material

Supplementary Figure 1 The ROC analysis results of top 10 most discriminant metabolites for distinguishing CRSwNP phenotypes. ROC, receiver operating characteristics; CRSwNP, chronic rhinosinusitis with nasal polyps; AUC, area under the curve.

Supplementary Figure 2 Relationship between eosinophilic infiltration and metabolites with good predictabilities. **(A–D)** Correlation between T-EOS percentage and citrulline, linoleic acid, adenosine and 4-guanidinobutyric acid. **(E–H)** Correlation between T-EOS percentage and citrulline, linoleic acid, adenosine and 4-guanidinobutyric acid. T-EOS, tissue eosinophil. Spearman correlation analysis was used.

Supplementary Table 1 ROC analysis results of top ten metabolites for discriminating eCRSwNP from neCRSwNP.
