# Supplementary figures and images for: The Role of Serum Metabolomics in Distinguishing Chronic Rhinosinusitis With Nasal Polyp Phenotypes
Source: Front Mol Biosci. 2021 Jan 12;7:593976. doi: 10.3389/fmolb.2020.593976 (PMC7835901; doi:10.3389/fmolb.2020.593976)

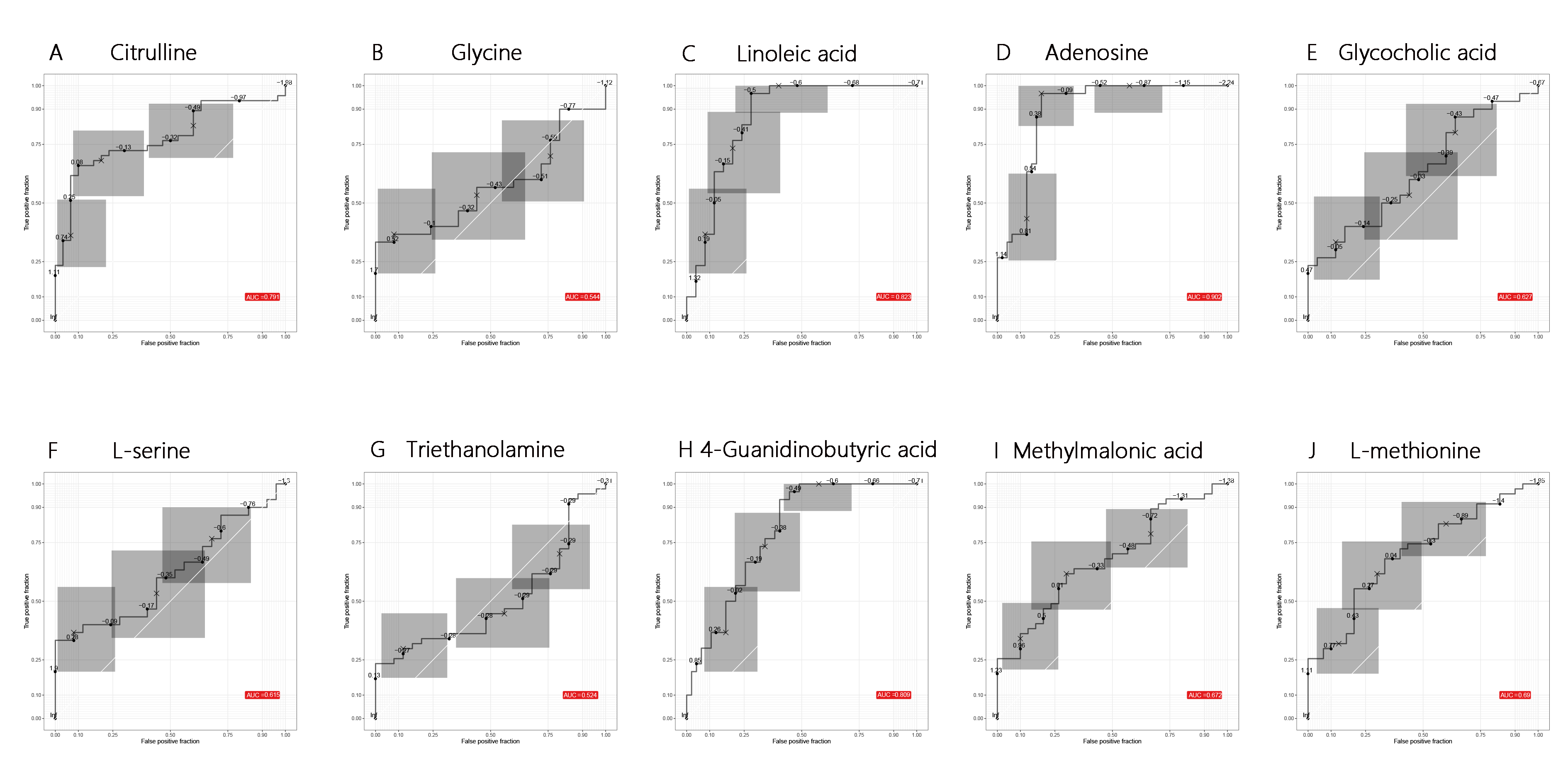

Supplement: Supplementary Figure 1 — The ROC analysis results of top 10 most discriminant metabolites for distinguishing CRSwNP phenotypes. ROC, receiver operating characteristics; CRSwNP, chronic rhinosinusitis with nasal polyps; AUC, area under the curve. [file Image_1.TIF]

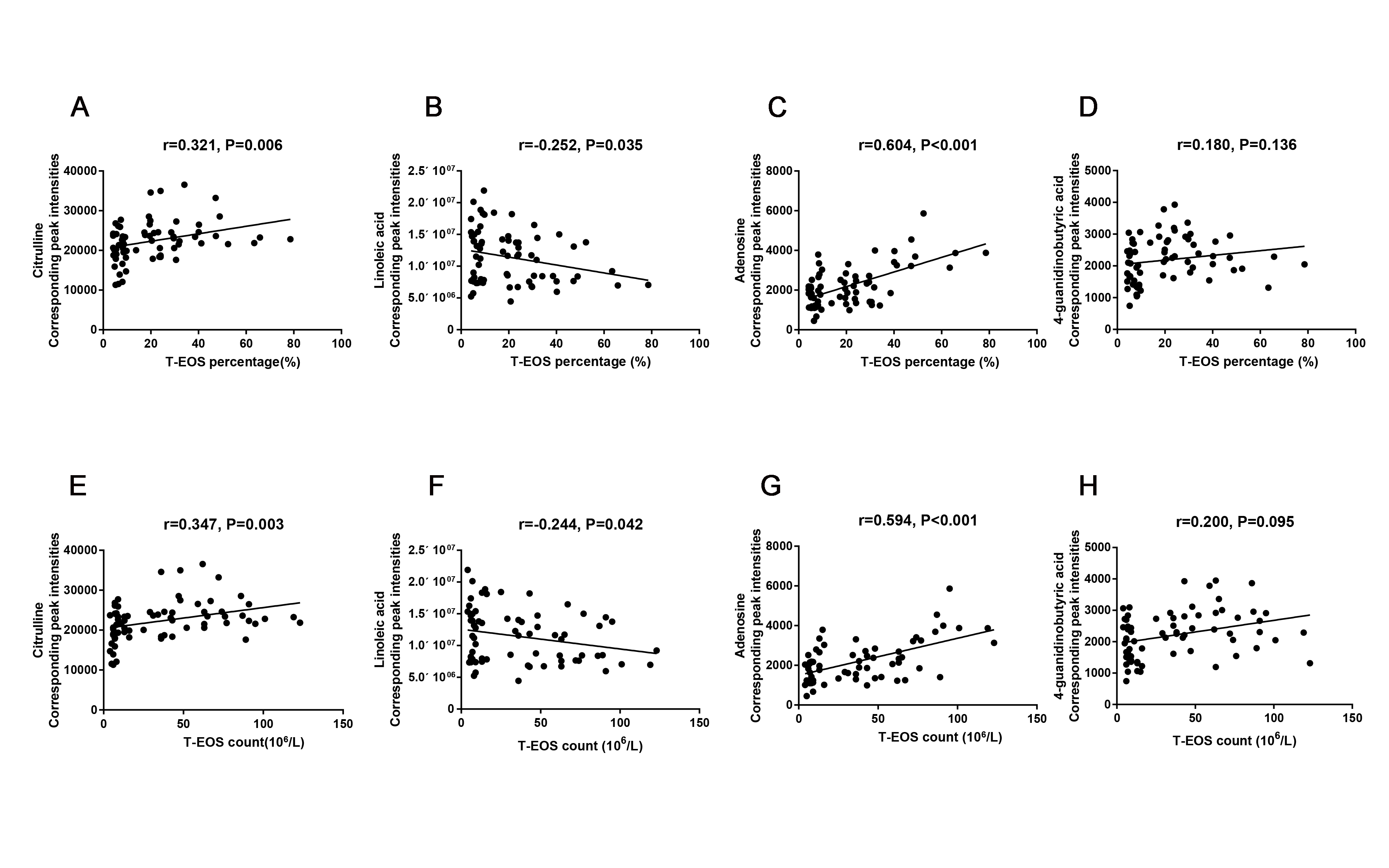

Supplement: Supplementary Figure 2 — Relationship between eosinophilic infiltration and metabolites with good predictabilities. (A–D) Correlation between T-EOS percentage and citrulline, linoleic acid, adenosine and 4-guanidinobutyric acid. (E–H) Correlation between T-EOS percentage and citrulline, linoleic acid, adenosine and 4-guanidinobutyric acid. T-EOS, tissue eosinophil. Spearman correlation analysis was used. [file Image_2.TIF]
