# Supplementary material for: The Role of Serum Metabolomics in Distinguishing Chronic Rhinosinusitis With Nasal Polyp Phenotypes
Source: Front Mol Biosci. 2021 Jan 12;7:593976. doi: 10.3389/fmolb.2020.593976 (PMC7835901; doi:10.3389/fmolb.2020.593976)
Supplement: Supplementary Table 1 — ROC analysis results of top ten metabolites for discriminating eCRSwNP from neCRSwNP. [file Table_1.DOCX]

| Metabolites | AUC | Standard error | 95% CI  lower upper | | Youden index  sensitivity specificity | |
| --- | --- | --- | --- | --- | --- | --- |
| Citrulline | 0.791 | 0.106 | 0.613 | 0.857 | 0.725 | 0.892 |
| Glycine | 0.544 | 0.139 | 0.427 | 0.704 | 0.614 | 0.637 |
| Linoleic acid | 0.823 | 0.097 | 0.679 | 0.901 | 0.973 | 0.784 |
| Adenosine | 0.902 | 0.084 | 0.731 | 0.970 | 0.957 | 0.813 |
| Glycocholic acid | 0.627 | 0.127 | 0.534 | 0.712 | 0.673 | 0.571 |
| L-serine | 0.615 | 0.118 | 0.486 | 0.733 | 0.591 | 0.589 |
| Triethanolamine | 0.524 | 0.133 | 0.421 | 0.628 | 0.912 | 0.232 |
| 4-Guanidinobutyric acid | 0.809 | 0.101 | 0.694 | 0.897 | 0.967 | 0.602 |
| Methylmalonic acid | 0.672 | 0.122 | 0.599 | 0.731 | 0.648 | 0.721 |
| L-methionine | 0.690 | 0.118 | 0.563 | 0.774 | 0.684 | 0.677 |

**Table S1.** ROC analysis results of top ten metabolites for discriminating eCRSwNP from neCRSwNP

eCRSwNP , eosinophilic chronic rhinosinusitis with nasal polyps; neCRSwNP, non-eosinophilic chronic rhinosinusitis with nasal polyps; CI, confidence interval; AUC, area under the curve
